# Fatal invasive cervical cancer secondary to untreated cervical dysplasia: a case report

**DOI:** 10.1186/1752-1947-5-316

**Published:** 2011-07-18

**Authors:** Stephan Braun, Daniel Reimer, Isolde Strobl, Ulrike Wieland, Petra Wiesbauer, Elisabeth Müller-Holzner, Siegfried Fessler, Arthur Scherer, Christian Marth, Alain G Zeimet

**Affiliations:** 1 Department of Obstetrics and Gynecology, Innsbruck Medical University, Anichstrasse 35, AT-6020 Innsbruck, Austria; 2 Institute of Virology, University of Cologne, Fürst-Prückler-Strasse 65, D-50935 Cologne, Germany; 3 Department of Obstetrics and Gynecology, Medical Services Hospital, Bressanone, Italy

## Abstract

**Introduction:**

Well-documented cases of untreated cervical intra-epithelial dysplasia resulting in fatal progression of invasive cervical cancer are scarce because of a long pre-invasive state, the availability of cervical cytology screening programs, and the efficacy of the treatment of both pre-invasive and early-stage invasive lesions.

**Case presentation:**

We present a well-documented case of a 29-year-old Caucasian woman who was found, through routine conventional cervical cytology screening, to have pathologic Papanicolaou (Pap) grade III D lesions (squamous cell abnormalities). She subsequently died as a result of human papillomavirus type 18-associated cervical cancer after she refused all recommended curative therapeutic procedures over a period of 13 years.

**Conclusion:**

This case clearly demonstrates a caveat against the promotion and use of complementary alternative medicine as pseudo-immunologic approaches outside evidence-based medicine paths. It also demonstrates the impact of the individualized demands in diagnosis, treatment and palliative care of patients with advanced cancer express their will to refuse evidence-based treatment recommendations.

## Introduction

Cases of intra-epithelial disease of the cervix are almost entirely attributable to human papillomavirus (HPV) infection. A minority of women exposed to HPV develop a persistent infection that affects the squamocolumnar junction where the ectocervix and endocervix meet. Within that junction, dynamic changes of the epithelium occur due to puberty, pregnancy, menopause and hormonal stimulation. The epithelium is vulnerable to noxae associated with smoking, contraceptive use and infection with other sexually transmitted diseases. Alterations of the epithelium are assessed by conventional cervical cytology screening and are scored according to either the Bethesda or the Papanicolaou system. The occurrence of reactive changes and/or cell abnormalities triggers either repetitions of the cytology screening to exclude temporary alterations or a cervical biopsy for histological diagnosis of cervical intra-epithelial neoplasia and cervical cancer. With the advent of HPV vaccination [1] and HPV screening [2] to identify women at risk of lesions with atypical or malignant cells prior to clinical manifestation, in current clinical practice a patient's HPV status should play a central role in the prevention of HPV-associated diseases [3].

Invasive cervical cancer has a long pre-invasive state, and cervical cytology screening programs are available. Moreover, HPV vaccination has been shown to be a successful tool of primary prevention [1], and treatment of pre-invasive lesions is effective. Invasive cancer is considered a preventable cancer in the so-called highly developed Western countries [3]. Consequently, invasive cancer of the cervix has become increasingly infrequent in this part of the world, but it remains a significant health problem in underdeveloped countries, where meticulous documentation of fatal courses of the disease plays a minor role. Thus, our knowledge of the lead time between dysplasia and the development of invasive cancer as well as progression from early-stage to metastasized cancer largely derives from extrapolating information from studies and textbooks, but very few case reports.

Herein we report a rather rare, yet well-documented case of a 29-year-old woman who, during the course of her disease, accepted multiple diagnostic procedures but refused any curative treatment beginning with the first assessment of cervical dysplasia and early-stage invasive cancer 10 years later. She finally refused to accept any interventional medical strategies, except for palliative care, at the stage of locally progressed and metastasized cervical cancer.

## Case presentation

A 29-year-old Caucasian woman was seen for her routine annual gynecologic examination, and conventional cytological screening of her cervix uteri revealed a pathologic finding scored as grade IIID under the Papanicolaou system. Repeat screening performed one year later revealed a grade IV pathologic finding, suggesting a high-grade squamous intra-epithelial lesion. Our patient refused the recommended diagnostic and therapeutic procedure of conization, and she was placed on a non-specified homeopathic therapy consisting of a vitamin C-containing regimen and subcutaneous administration of mistletoe lectins.

At the time of the first pathologic Papanicolaou test, our patient reported a normal menstrual cycle, no pregnancies, no use of oral contraceptives, no presence of any previous diseases or any surgery, no allergies, no smoking, and no use of illicit drugs. There was no evident lack in body hygiene. Except for her father's stomach cancer, her family and cancer-specific anamneses were unremarkable. On the basis of her grade V in Papanicoleau test, she was sent to our hospital's out-patient department.

A gynecologic examination at that time revealed obvious tumor growth confined to her cervix with no signs of extension to her vagina. A cervical biopsy showed moderately differentiated (tumor grade II) large-cell non-keratinizing squamous cell carcinoma of the cervix uteri. Neither lymphatic nor venous vascular space involvement was reported, but dense inflammatory cell infiltration of the tumor stroma was noted. Clinical staging was completed by cystoscopy, proctoscopy, and chest radiography (as allowed for accurate clinical staging by the International Federation of Gynecology and Obstetrics [FIGO]), which revealed stage IB2 cancer. Additional information was obtained by extended staging procedures, including computed tomographic (CT) and laparoscopic sampling of her para-aortic lymph nodes (the results of which would have had no influence on the assigned clinical stage according to FIGO guidelines).

However, our patient refused to undergo any further diagnostic procedures and instead underwent complementary medical treatment. This included regional hyperthermia, which led to her self-admission to a local hospital. She presented there with reduced physical status, large edema of the legs, and moist rales in her lungs. She also reported lower abdominal pain. Her clinical work-up revealed significant progression of her disease, which now included bilateral parametrial involvement, broad involvement of her dorsal bladder wall, infiltration of her outer rectum wall, pericardial and pleural effusion, bilateral hydronephrosis with laboratory signs of uremia (serum creatinine 17.4 mg/dL, serum uric acid 106 mg/dL), and tumor anemia with hemoglobin at 71 g/L. Two courses of hemodialysis were performed initially, followed by a right-sided nephrostomy after the failure of ureteral stenting due to tumor extension to her bladder. It was decided to commence hemodialysis on the basis of the patient's request for consequent evidence-based, palliative medical care after restoration of her renal function.

**Figure 1:**
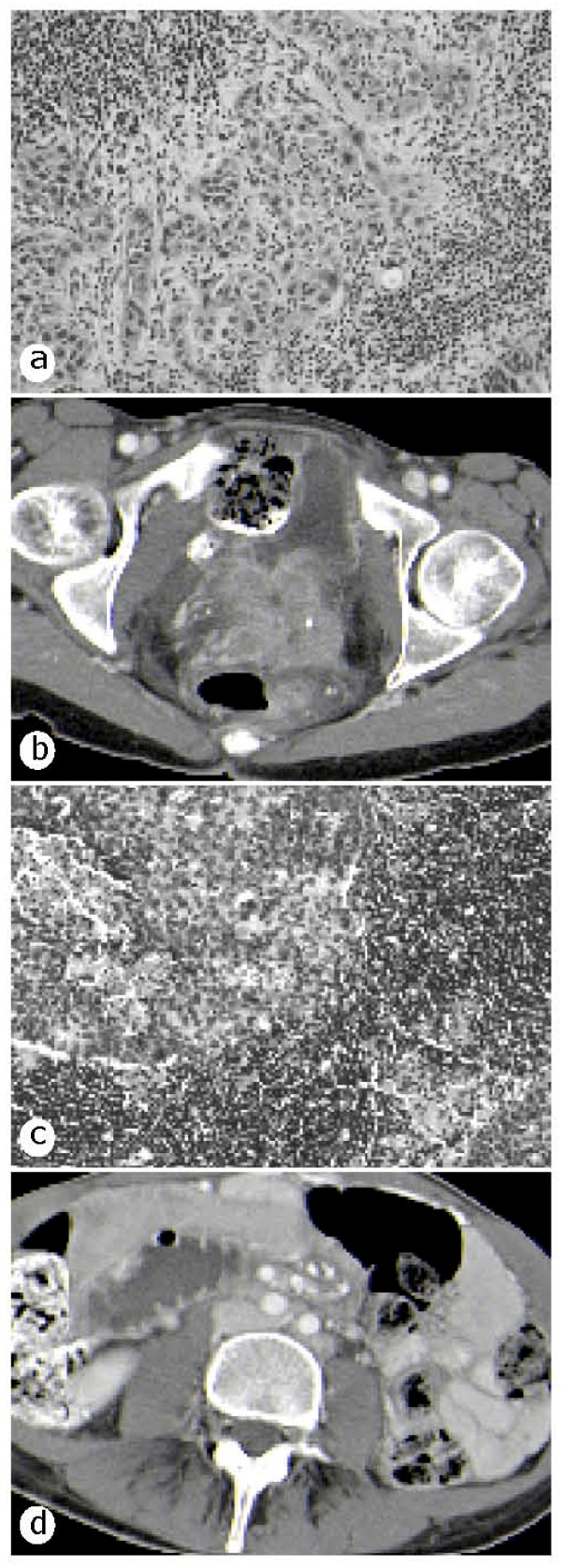
**Restaging of the tumor**. **(a) **formalin-fixed, paraffin-embedded biopsy of the invasive cervix cancer; **(b) **corresponding CT scan of the pelvis; **(c) **formalin-fixed, paraffin-embedded biopsy of the para-aortic lymph node metastases and her abdomen showing bladder invasion; and **(d) **corresponding CT scan of the enlarged para-aortic lymph nodes.

Restaging was performed, which indicated involvement of her bladder wall and adhesions to the ileocecal area (Figure 1b). All three para-aortic lymph nodes removed during re-laparoscopy were positive (Figures 1c and 1d), while no signs of distant metastasis were seen on the radiologic studies. The tumor was restaged to FIGO IVa, and concurrent cisplatin-based chemoradiation was recommended. Our patient, however, opted against our treatment recommendation and traveled to the Philippines for an alternative holistic treatment schedule involving several courses of Horvi-Reintoxin enzyme therapy, which consists of enzymatically processed snake poison that is purported to specifically inhibit glycosylation in tumor cells, thus conferring anti-tumoral activity.

**Figure 2:**
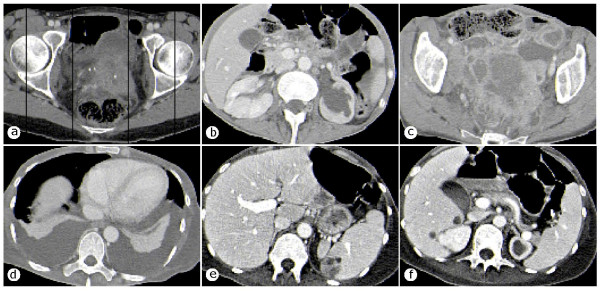
**CT scans**. **(a) **local tumor progression in her pelvis and vesical invasion and hemorrhage; **(b) **left ureter obstruction; **(c) **ileus through descending colon and rectosigmoid obstruction; **(d) **malignant pleural effusion; **(e) **spleen metastasis; **(f) **liver metastasis.

Our patient was repeatedly admitted to both Brixen and Innsbruck hospitals for erythrocyte transfusions because of spontaneous uterine hemorrhage and further local tumor progression (Figure 2a). Acute life-threatening hemorrhage prompted us to perform three sessions of arterial embolization: first, in both uterine arteries; second, in both internal pudendal arteries and re-embolization of her right uterine artery; and third, in her right superior and inferior vesical arteries and re-embolization of her left internal pudendic artery. In parallel, our patient continued her holistic alternative medical treatment, first with active fever treatment, during which pyrogenic lysates of bacteria were administered and second with combined application of Carnivora-Mistletoe-Ukrain (that is, capsules with plant extracts, subcutaneous injections of mistletoe lectins, capsules with extracts of celandine and *Chelidonium majus*), all of which are purported to have antitumoral activity.

Seven months later our patient was admitted to the hospital with clinical signs of chronic large bowel obstruction, and laparotomy and side-to-side ileoascendostomy became necessary, during which her left ureter (Figure 2b), descending colon, rectosigmoid and ileocecum appeared fixed by tumor masses and obstructed by large, lymphatic fluid-containing cysts (Figure 2c). Our patient overcame a postsurgical bowel paralysis and recovered fairly well. However, during the following days, palliative care was required for salvage from dyspnoea by bilateral pleuracentesis (Figure 2d), from mechanical and paralytic small and large bowel obstruction by distigmine bromide administration, and from recurrent visceral abdominal and neurogenic pain by morphine hydrochloride. On the basis of a CT scan, a paralytic ileus, together with metastasis to her spleen (Figure 2e) and her liver (Figure 2f), were diagnosed. One month later our patient died as a result of tumor cachexia, chronic small and large bowel ileus, septicemia and consecutive multiple organ failure. In her last will, she refused autopsy.

For HPV testing, we isolated DNA from the paraffin-embedded tumor shown in Figure 1a. With group-specific nested polymerase chain reactions for the detection of α-HPV DNA [4], we tested for the presence of 18 high-risk HPV types (16, 18, 26, 31, 33, 35, 39, 45, 51, 52, 53, 56, 58, 59, 66, 68, 73, and 82) and 18 low-risk HPV types (6, 11, 40, 42, 43, 44, 34, 54, 55, 57, 61, 70, 71, 72, 81, 83, 84, and 89). Our patient's tumor exclusively contained type 18 HPV DNA.

## Discussion

We have presented the case of a woman who had a pathologic cervical cytology screening at the age of 29 years, and died as a result of cervical cancer at the age of 42 after she had denied all recommended curative therapeutic procedures for 13 years. Except for the noted HPV type 18 infection, our patient's demographic characteristics included the typical socioeconomic and epidemiologic risk factors known for cervical cancer in that she was Caucasian, had a high socioeconomic status, reported no cigarette smoking, was nulliparous and reported no history of apparent promiscuity. It appears to be a rather rare case in terms of, on the one hand, accepting far-reaching diagnostic procedures such as laparoscopic lymph node sampling, and on the other, consistently refusing to accept all proposed evidence-based treatment recommendations over a total period of 13 years, with no psychological disorder being apparent.

Documented cases of untreated cervical dysplasia are rare, and ours appears to be only the second report published during the past 10 years that is retrievable in the currently available medical literature databases. The other case, reported in 2002, described a very short interval of one year between the diagnosis of cervical dysplasia and metastases in the bone, liver, and orbit [5]. This short interval between dysplasia and metastatic cervical cancer, however, raises questions as to whether the Pap smear was representative or whether invasive cancer was missed. Thus, our case presentation might be one of the very few examples of a complete clinical documentation of such "natural" progression among retrievable case reports in the medical literature.

Of note, we want to stress that the apparent inefficacy of the complementary alternative medical treatments practiced worldwide, which should have exerted a stimulatory effect on the immune system and hence an antineoplastic influence. Effects in preventing high-grade cervical lesions to date have been noted only for bivalent and quadrivalent vaccines against HPV type 16 or 18 and HPV types 6, 11, 16, and18, with vaccine efficacies well above 90% [1]. Remarkably, and in sharp contrast to the mentioned vaccine efficacy, the non-specific approaches used in complementary alternative medicine, as described in our present case, are deemed rather inefficacious.

HPV type 16 or 18 infections are responsible for approximately 60-80% of all invasive cancers, varying according to the patient's socioeconomic status [6]. Of all new HPV infections, both oncogenic and non-oncogenic type infections last between eight and five months, respectively, and the large majority of initially HPV-infected women show clearance within two years [7]. Pre-invasive surrogate lesions of squamous cervical cancer would be those of grade II and III, with the lowest potential of regression being that for grade III cervical intra-epithelial neoplasia [8]. Since our patient refused histopathological verification of the first cytological abnormalities in 1993, we were unable to determine whether a single, persistent HPV type 18 infection gave rise to her cervical cancer, which was diagnosed in 2003. The assumption that this was the case is highly likely to be true, since progression from HPV infection to invasive cancer is believed to take place during the course of several years, although we cannot exclude HPV type 18 reinfection after initial clearance. Cervical precursor lesions of oncogenic HPV infections, such as HPV type 18 in our case, are known to persist longer and progress more often than non-oncogenic type infections [9]. The likelihood of regression, stable dysplasia or progression from moderate cervical dysplasia (CIN II, which could have been the underlying disease in our patient, who had Pap IIID and Pap IV) is known to be almost equal. Because progression lead times are usually in the two- to five-year range [10], even if we take into account a potential reinfection as well as some time for progression from the first invasion to the bulky disease (on which we have no firm information available), in our case a gradual escape of the tumor from the host's immune surveillance may explain the rather slow progression to bulky cervix cancer over a ten-year period.

This ten-year period of uninfluenced tumor growth also allowed for systemic spread and a pattern of distant metastasis that, to the best of our knowledge, has not thus far been reported in the literature, but suggests a much more complex homing pattern of disseminated tumor cells. Overall, cervical cancer has a low propensity for distant hematogenous metastatic spread. The first clinical sign of metastasis to para-aortic lymph nodes, that is, beyond the true pelvis, was assessed after 10 years on the basis of a CT scan. Furthermore, the most common sites of distant metastasis are the lung, liver, bone and, rarely, the peritoneum. Single reports would add the orbit [5] and bone marrow [11]. Our patient's liver and spleen metastasis as well as carcinosis peritonei shortly before her death are rarely seen, but further contribute to our knowledge of viable tumor cell spread in cervical cancer.

## Conclusion

In summary, we have presented an unusual case of untreated, presumably HPV type 18-induced cervical dysplasia with progression to invasive and finally metastatic cervical cancer that demonstrated a ten-year lead time between the diagnosis of dysplasia and invasive cancer. This case serves as a caveat against the promotion and use of complementary alternative medicine as pseudo-immunologic approaches outside evidence-based medicine paths. It also highlights the individualized demands in diagnosis, treatment and palliative care of advanced cancer patients who express their will to refuse evidence-based treatment recommendations.

## Consent

Written informed consent for publication could not be obtained despite all reasonable attempts to trace the patient's family. Every effort was made to protect the identity of our patient, and there is no reason to believe that any of her relatives would object to publication.

## Competing interests

The authors declare that they have no competing interests.

## Authors' contributions

IS, PW, SF, AS and CM cared for the patient during her time in the hospital (LKH Innsbruck and LKH Brixen) and assisted in data collection and the preparation of the manuscript. SB, DR and AGZ were the major contributors in writing the manuscript. EMH performed the histological examination of the tumor tissues. UW performed the HPV testing. All authors read and approved the final manuscript.
